# Efficacy and safety of bevacizumab biosimilar compared with reference bevacizumab in locally advanced and advanced non-small cell lung cancer patients: A retrospective study

**DOI:** 10.3389/fonc.2022.1036906

**Published:** 2023-01-09

**Authors:** Zhiting Zhao, Luqing Zhao, Guohao Xia, Jianwei Lu, Bo Shen, Guoren Zhou, Fenglei Wu, Xiao Hu, Jifeng Feng, Shaorong Yu

**Affiliations:** ^1^ Department of Medical Oncology, The Affiliated Cancer Hospital of Nanjing Medical University & Jiangsu Cancer Hospital & Jiangsu Institute of Cancer Research, Nanjing, Jiangsu, China; ^2^ Department of Oncology, The Affiliated Suqian First People's Hospital of Nanjing Medical University & Suqian First Hospital, Suqian, Jiangsu, China; ^3^ Department of Oncology, The Affiliated Hospital of Kangda College of Nanjing Medical University & The First People’s Hospital of Lianyungang, Lianyungang, Jiangsu, China

**Keywords:** antiangiogenic treatment, bevacizumab, biosimilar, non-small cell lung cancer, lung cancer

## Abstract

**Background:**

Bevacizumab has played an important role in the systemic treatment of patients with advanced non-small-cell lung cancer (NSCLC) without gene mutation. In recent years, bevacizumab biosimilar has received marketing approval based on the results of phase III clinical studies. However, more clinical data are needed to verify the efficacy and safety of bevacizumab biosimilar in clinical application.

**Materials and methods:**

We identified 946 patients with locally advanced or metastatic NSCLC who were treated with bevacizumab biosimilar or bevacizumab from January 1, 2019 to November 30, 2021. Comparisons and statistical analyses of bevacizumab biosimilar and bevacizumab were made in terms of efficacy and safety. Efficacy evaluation was performed directly in accordance with RECIST v1.1. Adverse events were graded following the National Cancer Institute Common Terminology Criteria for Adverse Events v5.0.

**Results:**

The objective response rates (ORRs) were 28.9% in the biosimilar group (n=551) and 30.9% in the reference group (n=395; unstratified ORR risk ratio: 0.934, 95% confidence interval [CI]: 0.677–1.138; unstratified ORR risk difference: −0.020, 95% CI: −0.118–0.035). The estimated median progression-free survival (mPFS) were 6.27 (95% CI: 5.53–7.01) and 4.93 (95% CI: 4.24–5.62) months in the biosimilar and reference groups, respectively (*P*=0.296). The number of treatment lines, combined treatment regimens and with or without radiotherapy were significant factors affecting the PFS of both groups (*P*<0.001, *P*=0.001, *P*=0.039). Different genetic mutations and dose intensity were not the main factors affecting PFS (*P*=0.627, *P*=0.946). The incidences of treatment-emergent adverse events (TEAEs) were 76.41% in the biosimilar group and 71.65% in the reference group (*P*=0.098). The incidences of grade 3 or higher TEAEs were 22.14% and 19.49% in the biosimilar and reference groups, respectively (*P*=0.324).

**Conclusions:**

Bevacizumab biosimilar is equivalent in efficacy to bevacizumab in patients with locally advanced and advanced NSCLC. It showed acceptable toxicity profile and no new adverse events. Patients who were excluded by clinical trials can also benefit from bevacizumab biosimilar.

## Introduction

Lung cancer is the leading cause of cancer-related deaths worldwide with an estimated 1.8 million deaths each year (1). Approximately 57% of patients have distant metastasis at the time of initial diagnosis and lose surgical indications (2). Therefore, systemic therapy plays an important role in lung cancer treatment. Vascular endothelial growth factor (VEGF) acts as a key regulator of tumor angiogenesis and is associated with increased risks of recurrence, metastasis, and death (3, 4). Bevacizumab is a recombinant humanized monoclonal antibody that specifically interrupts the interaction between human VEGF and endothelial cell surface receptors, inhibiting the biological activity of VEGF and limiting angiogenesis (5). Randomized controlled trials have demonstrated that bevacizumab in combination with chemotherapy improves overall survival (OS) and progression-free survival (PFS) relative to chemotherapy alone (6, 7). In addition, recent evidence points to novel combinations of bevacizumab with another targeted therapy or immunotherapy, as well as maintenance therapy after disease progression (8).

A biosimilar is a biological product that is highly similar to the reference product with no clinically remarkable differences in safety, purity, or potency (9). A biosimilar is an important avenue to reduce patient expenditure and the financial burden of national healthcare systems while maintaining therapeutic effect (10). However, a biosimilar is not an exact copy of its reference product and thus requires vigilance and concern in clinical application (11, 12).

Currently, prospective clinical trials have confirmed that bevacizumab biosimilar in combination with platinum-containing two-drug chemotherapy has similar efficacy and safety compared with bevacizumab in patients with untreated advanced non-squamous non-small-cell lung cancer (NSCLC) (13, 14). In 2019, China National Medical Products Administration approved the marketing of the bevacizumab biosimilar developed by Qilu Pharmaceutical Co., Ltd. (trade name: Encoda) with all indications approved for bevacizumab in China. However, no clinical data has verified the efficacy and safety of this biosimilar in clinical application. Existing trials (15–17) have excluded patients receiving previous treatment, patients receiving combination with targeted therapy or immunotherapy, patients with brain metastases, patients with rare genetic mutations (such as EML4-ALK rearrangement), and patients with Eastern Cooperative Oncology Group (ECOG) scores greater than 2. The efficacy and safety of bevacizumab biosimilar have no concensus in these populations. The purpose of the current investigation was to retrospectively analyze and compare the efficacy and safety of bevacizumab biosimilar and reference bevacizumab in patients with locally advanced and advanced NSCLC in clinical application and to provide reference for clinical decision-making.

## Materials and methods

We studied the medical records of all patients with locally advanced and advanced non-squamous NSCLC treated at Jiangsu Cancer Hospital from January 1, 2019 to November 30, 2021. We screened patients who received bevacizumab biosimilar or bevacizumab treatment. All patients included in the study had at least one measurable disease. This study was approved by the Academic Ethics Committee of Jiangsu Cancer Hospital (reference No.036 (2022)). All patients gave informed consent and signed the consent form.

### Data collection and response assessment

Medical records were examined and separated by clinical pathologic features and treatment histories. Radiographic examinations were performed to assess the efficacy at two cycles after initiation, and then the state of the tumor was assessed every two cycles or when symptoms of suspected disease progression occurred. Data and follow-up records were updated as of December 1, 2021. Efficacy evaluation was performed directly in accordance with the Response Evaluation Criteria in Solid Tumor 1.1. The best response to bevacizumab biosimilar or bevacizumab treatment was defined as a complete response (CR) or partial response (PR) and stable disease achieved at least once during therapy. The primary efficacy endpoint was the objective response rate (ORR) defined as the CR or PR rate. The secondary efficacy endpoint was the progression-free survival (PFS) defined as the time from treatment initiation to clinical or radiographic progression or death. Adverse events (AEs) were graded by the National Cancer Institute Common Terminology Criteria for Adverse Events v5.0.

### Statistical analysis

Differences in baseline clinical features, efficacy, and safety between groups were measured by χ^2^ test or independent T test. Survival data were estimated using the Kaplan–Meier method, including 95% confidence intervals (CIs). Significant differences between these curves were determined using log-rank test. Multivariate analysis of PFS was conducted by Cox proportional risk analysis. All statistical analyses were conducted using the SPSS (version 25.0) and R software (version 3.6.3). Statistical significance was set at *P*<0.05.

## Results

### Patient characteristics and treatment exposure

A total of 946 patients were included in this study, including 551 patients who received bevacizumab biosimilar (biosimilar group) and 395 patients who received bevacizumab (reference group). The baseline characteristics of the patients are summarized in **Table 1**. The subjects’ demographics and baseline disease characteristics were well balanced between the treatment groups with no statistical differences. Overall, the median age of the 946 patients was 60.5 years, including 533 males (56.34%) and 413 females (43.66%). Adenocarcinoma was the most common pathological type (98.52%). The remaining 14 nonadenocarcinomas included 7 adenosquamous carcinoma, 3 sarcomatoid carcinoma, 2 large cell carcinoma, and 2 undifferentiated carcinoma. The proportion of patients without gene mutation was the highest (44.83%), followed by patients with EGFR L858R mutation (22.69%) and patients with EGFR exon 19 deletion (17.12%). Before initial treatment with bevacizumab or bevacizumab biosimilar, 209 patients (21.88%) had brain metastases, 136 patients (14.38%) had liver metastases, and 136 patients (14.38%) had clinically diagnosed hypertension.

**Table 1:** Clinical characteristics and treatment exposure of patients.

	Total (n=946)	biosimilar group (n=551)	reference group (n=395)	P
Sex				
Male	533 (56.34%)	305 (55.35%)	228 (57.72%)	0.469
Female	413 (43.66%)	246 (44.65%)	167 (42.48%)	
Age (years)				
≥60	524 (55.39%)	303 (54.99%)	221 (55.95%)	0.770
<60	422 (44.61%)	248 (45.01%)	174 (44.05%)	
Pathological type				
adenocarcinoma	932 (98.52%)	543 (98.55%)	389 (98.48%)	0.933
Nonadenocarcinoma	14 (1.48%)	8 (1.45%)	6 (1.52%)	
Stage of cancer				
IIIB	54 (5.71%)	32 (5.81%)	22 (5.57%)	0.876
IV	892 (94.29%)	519 (94.19%)	373 (94.43%)	
Gene mutation type				
No genetic mutation	448 (48.41%)	247 (44.83%)	201 (50.89%)	0.066
EGFR exon 18 point mutation	4 (0.42%)	2 (0.36%)	2 (0.51%)	0.738
EGFR exon 19 deletion	162 (17.12%)	101 (18.33%)	61 (15.44%)	0.245
EGFR exon 20 insertion	24 (2.54%)	15 (2.72%)	9 (2.28%)	0.669
EGFR the L858R point mutation	209 (21.04%)	125 (22.69%)	84 (21.27%)	0.604
EGFR double mutation	10 (1.06%)	4 (0.73%)	6 (1.52%)	0.335
ALK rearrangement	26 (2.75%)	14 (2.54%)	12 (3.04%)	0.645
ROS1 rearrangement	8 (0.85%)	6 (1.09%)	2 (0.51%)	0.480
RET rearrangement	7 (0.74%)	6 (1.09%)	1 (0.25%)	0.249
BRAF mutation	6 (0.63%)	4 (0.73%)	2 (0.51%)	1.000
HER2 mutation	9 (0.95%)	6 (1.09%)	3 (0.76%)	0.742
KRAS mutation	26 (2.75%)	17 (3.09%)	9 (2.28%)	0.454
MET aberration	7 (0.74%)	4 (0.73%)	3 (0.76%)	1.000
with brain metastases				
Yes	209 (21.88%)	120 (21.78%)	89 (22.03%)	0.783
No	737 (78.12%)	431 (78.22%)	306 (77.97%)	
with liver metastases				
Yes	136 (14.38%)	72 (13.07%)	64 (16.20%)	0.175
no	810 (85.62%)	479 (86.93%)	331 (83.80%)	
with a history of hypertension				
yes	136 (14.38%)	81 (14.70%)	55 (13.92%)	0.737
no	810 (85.62%)	470 (85.30%)	340 (86.08%)	
**the mean duration of exposure (m)**	7.54	6.98	8.31	0.121
**the mean number of doses**	7.64	7.53	7.81	0.364
**the mean dose intensity (mg/kg)**	8.07	8.37	7.64	0.823

Exposure to treatment agents was comparable between the groups. The mean durations of exposure were 6.98 (standard deviation [SD], 6.03) and 8.31 (9.74) months (*P*=0.121) and the mean number of doses were 7.5 (5.45) and 7.8 (6.02, *P*=0.364) in the biosimilar and reference groups, respectively. The mean dose intensities in the biosimilar and reference groups were 8.37 and 7.64 mg/kg per cycle, respectively (*P*=0.823).

### Efficacy

**Figure 1:**
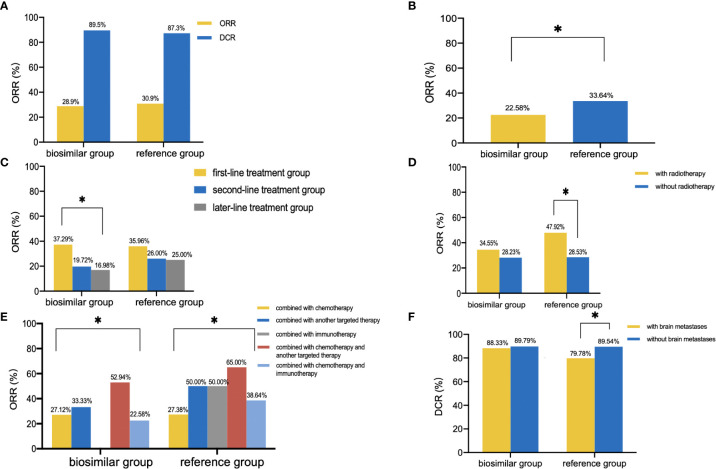
Clinical outcomes. **(A)**, ORR and DCR of the biosimilar and reference groups. **(B)**, ORR of patients treated with bevacizumab (or biosimilar) in combination with chemotherapy and immunotherapy. **(C)**, ORR of different number of treatment lines in the two groups. **(D)**, ORR of patients with or without radiotherapy in the two groups. **(E)**, ORR of different combined treatment regimens in the two groups. **(F)**, DCR of patients with or without brain metastases in the two groups. ^*^ *P*<0.05.

No patient achieved CR. In the biosimilar group (n=551), 159 patients experienced PR and 334 patients experienced SD with an ORR of 28.9% (95% CI: 25.1%–32.7%) and a disease control rate (DCR) of 89.5% as shown in **Figure 1A**. In the reference group (n=395), 122 patients developed PR and 223 patients developed SD with an ORR of 30.9% (95% CI: 26.3%–35.5%) and a DCR of 87.3%. As is shown in **Table 2**, the unstratified ORR risk ratio was 0.934 with a 95% CI of 0.767–1.138 and a 90% CI of 0.792–1.103. The unstratified ORR risk difference was −0.020 with a 95% CI of −0.118–0.035 and a 90% CI of −0.105–0.023. The results fell within the range prescribed by the Food and Drug Administration (FDA), Japan’s Pharmaceuticals and Medical Devices Agency (PMDA), and the European Medicines Agency (EMA). This result indicates that the bevacizumab biosimilar showed similar efficacy to bevacizumab. As shown in **Table 3**, the subgroup analyses were performed based on the following subgroups: sex, age, pathology, stage, number of treatment lines, radiotherapy, combined treatment regimens, combined chemotherapy regimens (if combined with chemotherapy), dose intensity, genetic mutations, brain metastasis, liver metastasis, and history of hypertension. In a subgroup analysis of combined treatment regimens, the ORR of the biosimilar group was lower than that of the reference group when combined with chemotherapy and immunotherapy (22.58% vs. 33.64%, *P*=0.048, **Figure 1B**). Further logistic regression analysis showed that the influencing factors of ORR in patients under the biosimilar combined with chemotherapy and immunotherapy were the number of treatment lines and combination with radiotherapy (*P*=0.021, 0.008). Product type (i.e., bevacizumab or bevacizumab biosimilar) was not a main factor (*P*=0.604). Overall, patients in the first-line treatment group revealed relatively higher ORR and DCR than those in the second- or later-line therapy (biosimilar group: ORR: 37.29% vs. 19.72% vs. 16.98%, *P*<0.001, **Figure 1C**; DCR: 93.07% vs. 85.92% vs. 83.96%, *P*=0.009; reference group: ORR: 35.96% vs. 26.00% vs. 25.00%, *P*=0.080; DCR: 93.10% vs. 82.00% vs. 80.43%, *P*=0.002). Patients with combined radiotherapy showed higher ORR than those without combined radiotherapy (biosimilar group: 34.55% vs. 28.23%, *P*=0.326; reference group: 47.92% vs. 28.53%, *P*=0.006; **Figure 1D**). Remarkable differences in ORR and DCR were observed in the different combination groups as shown in **Figure 1E**. Patients in the combination chemotherapy and another targeted therapy had the highest ORR (56.34%) and patients in the combination of another targeted therapy had the highest DCR (92.50%). In addition, patients without brain metastases showed higher DCR than patients with brain metastases (84.69% vs. 89.69%, *P*=0.045, **Figure 1F**).

**Table 2:** Comparison of overall response rate between biosimilar and reference groups.

	biosimilar group (n=551)	reference group (n=395)
Best overall response, n (%)		
PR	159 (28.9%)	122 (30.9%)
SD	334 (60.6%)	223 (56.4%)
PD	58 (10.5%)	50 (12.7%)
ORR	28.9%	30.9%
95% exact CI	25.1%-32.7%	26.3%-35.5%
Treatment comparison (vs referrence bevacizumab group)		
**Unstratified ORR risk ratio**	0.934	
95% CI of risk ratio	0.767-1.138	
90% CI of risk ratio	0.792-1.103	
**Unstratified ORR risk difference**	-0.020	
95% CI of difference	-0.118-0.035	
90% CI of difference	-0.105-0.023	

ORR, overall/objective response rate; PR, partial response; SD, stable disease; PD, progressive disease.

**Table 3:** Subgroup analysis of ORR and DCR between and within biosimilar and reference groups.

	ORR(%)					DCR(%)				
Variable	biosimilar group	p^1^	reference group	p^2^	p^3^	biosimilar group	p^1^	reference group	p^2^	p^3^
Sex										
male (n=533)	29.18%	0.852	31.58%	0.728	0.551	88.52%	0.419	85.09%	0.115	0.244
female (n=413)	28.46%		29.94%		0.744	90.65%		90.42%		0.937
Age (years)										
≥60 (n=524)	27.72%	0.516	30.77%	0.955	0.448	88.78%	0.557	88.69%	0.365	0.974
<60 (n=422)	30.24%		31.03%		0.862	90.32%		85.63%		0.954
Pathological type										
adenocarcinoma (n=932)	28.55%	0.349	31.11%	0.753	0.399	89.32%	1.000	87.66%	0.169	0.432
nonadenocarcinoma (n=14)	50.00%		16.67%		0.301	100.00%		66.67%		0.165
Stage of cancer										
IIIB (n=54)	34.38%	0.478	36.36%	0.567	0.880	87.50%	0.938	90.91%	0.851	1.000
IV (n=892)	28.52%		30.56%		0.508	89.60%		87.13%		0.254
Number of treatment lines										
1 (n=506)	37.29%	0.000*	35.96%	0.080	0.760	93.07%	0.009*	93.10%	0.002*	0.988
2 (n=242)	19.72%		26.00%		0.248	85.92%		82.00%		0.410
≥3 (n=198)	16.98%		25.00%		0.165	83.96%		80.43%		0.516
Combined with radiotherapy										
with (n=103)	34.55%	0.326	47.92%	0.006*	0.168	90.91%	0.715	95.83%	0.059	0.445
without (n=843)	28.23%		28.53%		0.923	89.31%		86.17%		0.166
Combined treatment regimens										
combined with chemotherapy (n=690)	27.12%	0.001*	27.38%	0.003*	0.939	88.77%	0.068	87.38%	0.387	0.575
combined with another targeted therapy (n=40)	33.33%		50.00%		0.602	94.44%		75.00%		0.277
combined with immunotherapy (n=8)	0.00%		50.00%		0.250	50.00%		50.00%		1.000
combined with chemotherapy and another targeted therapy (n=71)	52.94%		65.00%		0.357	92.16%		90.00%		1.000
combined with chemotherapy and immunotherapy (n=137)	22.58%		38.64%		0.048*	91.40%		88.64%		0.756
Combined chemotherapy regimens (if combined)										
combined with pemetrexed (n=26)	10.00%	0.358	12.50%	0.609	1.000	70.00%	0.180	81.25%	0.148	0.508
combined with taxane (n=46)	16.67%		25.00%		0.698	86.67%		68.75%		0.241
combined with gemcitabine (n=9)	25.00%		40.00%		1.000	75.00%		80.00%		1.000
combined with pemetrexed–platinum (n=474)	29.84%		27.78%		0.621	90.31%		89.35%		0.731
combined with taxane–platinum (n=95)	24.14%		32.43%		0.377	89.66%		89.19%		0.942
Dose intensity										
low-dose (n ≤ 7.5mg/kg) (n=461)	33.19%	0.061	29.79%	0.567	0.432	92.48%	0.055	88.09%	0.590	0.112
high-dose (7.5mg/kg<n ≤ 15.0mg/kg) (n=485)	25.85%		32.50%		0.125	87.38%		86.25%		0.727
Gene mutation type										
No genetic mutation (n=448)	26.32%	0.196	32.84%	0.165	0.131	87.45%	0.169	90.55%	0.190	0.225
EGFR exon 18 point mutation (n=4)	100.00%		0.00%		0.333	100.00%		100.00%		NA
EGFR exon 19 deletion (n=162)	23.76%		24.59%		0.905	84.16%		88.52%		0.440
EGFR exon 20 insertion (n=24)	46.67%		22.22%		0.389	93.33%		66.67%		0.130
EGFR the L858R point mutation (n=209)	35.20%		29.76%		0.412	90.20%		79.38%		0.057
EGFR double mutation (n=10)	50.00%		0.00%		0.133	100.00%		83.33%		1.000
ALK rearrangement (n=26)	28.57%		41.67%		0.683	100.00%		91.67%		0.462
ROS1 rearrangement (n=8)	33.33%		100.00%		0.429	83.33%		100.00%		1.000
RET rearrangement (n=7)	33.33%		100.00%		0.429	100.00%		100.00%		NA
BRAF mutation (n=6)	0.00%		0.00%		NA	100.00%		100.00%		NA
HER2 mutation (n=9)	33.33%		66.67%		0.524	66.67%		100.00%		0.500
KRAS mutation (n=26)	29.41%		44.44%		0.667	94.12%		100.00%		1.000
MET aberration (n=7)	0.00%		0.00%		NA	100.00%		100.00%		NA
Brain metastases										
with (n=209)	26.67%	0.549	23.60%	0.091	0.614	88.33%	0.645	79.78%	0.015*	0.089
without (n=737)	29.47%		33.01%		0.306	89.79%		89.54%		0.913
Liver metastases										
with (n=136)	23.61%	0.292	28.13%	0.601	0.548	86.11%	0.319	87.50%	0.967	0.926
without (n=810)	29.65%		31.42%		0.589	89.98%		87.31%		0.235
A history of hypertension										
with (n=136)	30.86%	0.666	40.00%	0.115	0.272	91.36%	0.550	90.91%	0.391	0.928
without (n=810)	28.51%		29.41%		0.780	89.15%		86.76%		0.300

*: p<0.05; ORR, overall/objective response rate; DCR, disease control rate;p1, hypothesis testing parameters within the biosimilar group;p2, hypothesis testing parameters within the referrence group;p3, hypothesis testing parameters between the biosimilar group and the reference group

**Figure 2:**
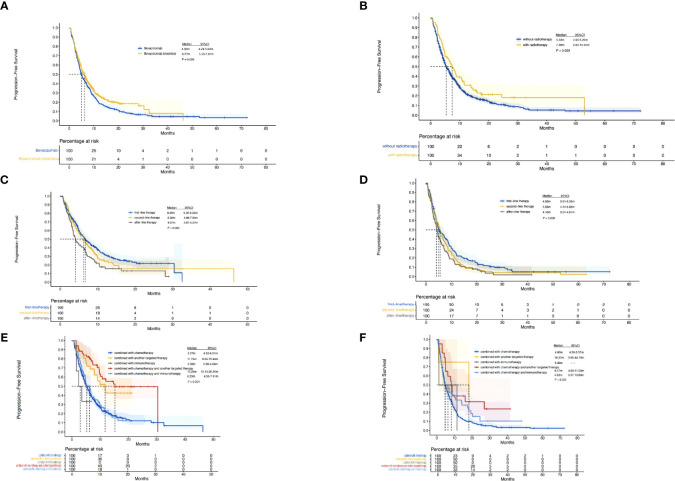
Kaplan–Meier curves. **(A)**, PFS of the biosimilar and reference groups. **(B)**, PFS of all the patients with or without radiotherapy. **(C)**, PFS of patients in the biosimilar group with different number of treatment lines. **(D)**, PFS of patients in the reference group with different number of treatment lines. **(E)**, PFS of patients in the biosimilar group with different combined treatment regimens. **(F)**, PFS of patients in the reference group with different combined treatment regimens.

**Figure 3:**
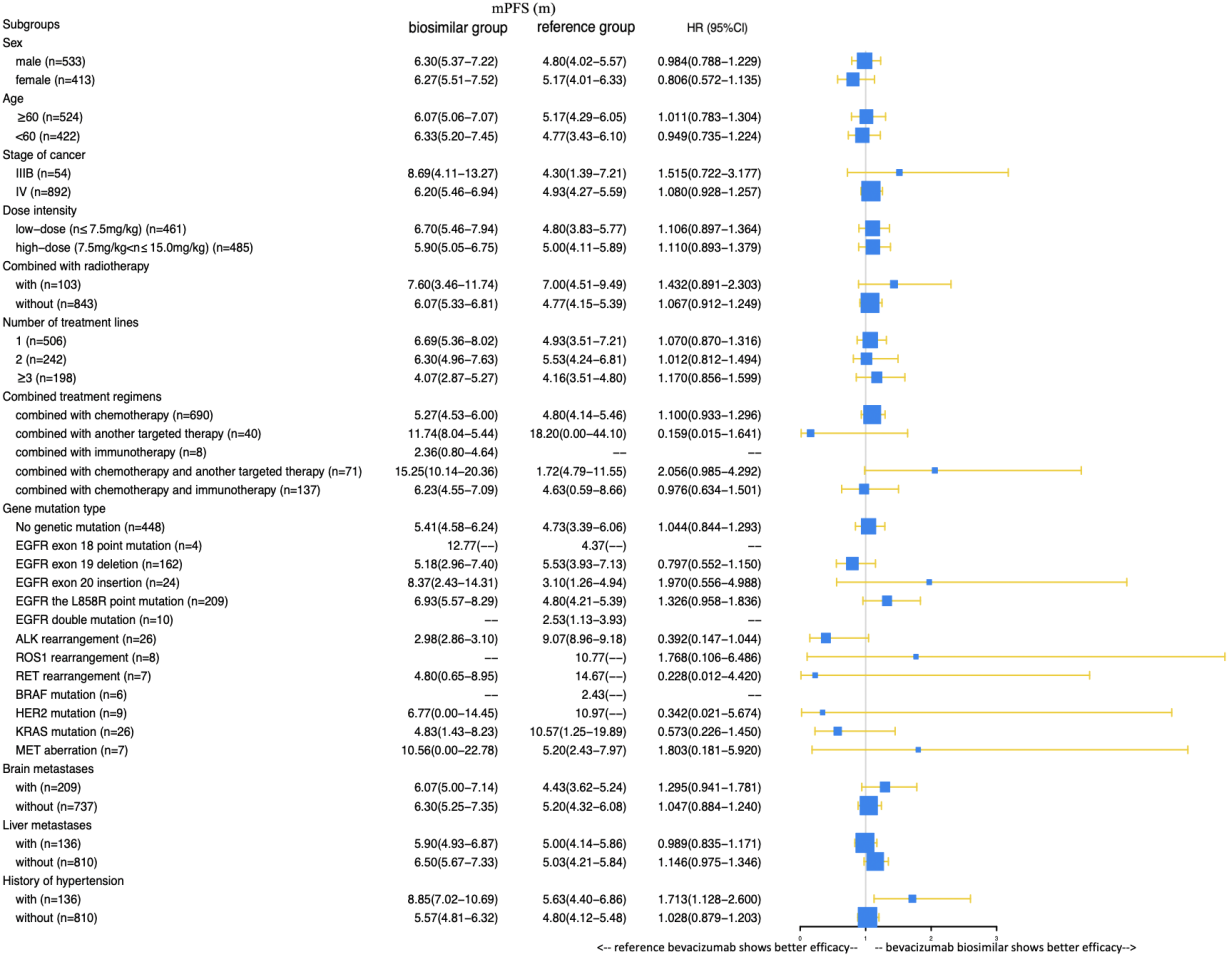
Comparison of the effects of bevacizumab biosimilar and reference bevacizumab on the PFS of each subgroup.

A total of 385 (69.9%) patients progressed or died in the biosimilar group compared with 363 (91.9%) patients in the reference group. Based on the Kaplan–Meier analysis, the estimated median PFS (mPFS) values were 6.27 (95% CI: 5.53–7.01) months in the biosimilar group and 4.93 (95% CI: 4.24–5.62) months in the reference group as shown in **Figure 2**. Based on the Cox regression model, the estimated hazard ratio (HR) for bevacizumab biosimilar and bevacizumab comparison was 1.084 (95% CI: 0.932–1.260, *P*=0.296). The analyses showed that the long-term efficacies of the two treatment groups were similar. Overall, the mPFS was 5.53 months (95% CI: 4.98–6.09) for all patients. The number of treatment lines, radiotherapy, and combined treatment regimens were statistically significant for PFS as shown in **Table 4**. In addition, based on the Cox regression analysis of the subgroups, no statistical difference was observed in the PFS of the bevacizumab biosimilar and bevacizumab groups except for the subgroup with a history of hypertension as shown in **Figure 3**. In a subgroup of patients with a history of hypertension, bevacizumab biosimilar obtained longer PFS than reference bevacizumab (8.85 vs. 6.63 months, *P*=0.047). The mean dose intensity of the biosimilar group was significantly higher than that of the reference group (8.28 vs. 7.37 mg/kg, *P*=0.007) in this subgroup. Based on multivariate analysis, the product type was not the main factor affecting PFS (*P*=0.595).

**Table 4:** Univariate analysis and multivariate analysis of factors affecting the progression-free survival (PFS) of all patients.

	Univariate analysis		Multivariate analysis	
	HR (95%Cl)	P value	HR (95%Cl)	P value
Sex				
male vs female	0.978 (0.846-1.130)	0.759		
Age				
≥60 years vs <60 years	1.013 (0.877-1.171)	0.862		
Pathological type				
adenocarcinoma vs nonadenocarcinoma	0.923 (0.494-1.723)	0.801		
Stage of cancer				
IIIB vs IV	0.777 (0.558-1.081)	0.135		
Product				
Bevacizumab biosimilar vs reference Bevacizumab	1.226 (1.061-1.417)	0.006	1.084 (0.932-1.260)	0.296
Dose intensity				
low-dose vs high-dose	0.995 (0.862-1.149)	0.946		
Combined with radiotherapy				
yes vs no	0.743 (0.587-0.940)	0.014	0.778 (0.613-0.987)	0.039*
Number of treatment lines				
1.000	1.000	0.000	1.000	0.001*
2.000	1.174 (0.988-1.394)		1.139 (0.957-1.355)	
≥3	1.485 (1.237-1.7782)		1.407 (1.168-1.695)	
Combined treatment regimens				
combined with chemotherapy	1.000	0.000	1.000	0.000*
combined with another targeted therapy	0.417 (0.617-3.089)		0.446 (0.284-0.700)	
combined with immunotherapy	1.381 (0.267-0.519)		1.260 (0.559-2.841)	
combined with chemotherapy and another targeted therapy	0.372 (0.267-0.519)		0.399 (0.295-0.560)	
combined with chemotherapy and immunotherapy	0.847 (0.686-1.046)		0.835 (0.673-1.036)	
Combined chemotherapy regimens (if combined)				
combined with pemetrexed	1.000	0.068		
combined with taxane	1.484 (0.876-2.514)			
combined with gemcitabine	1.497 (0.665-3.368)			
combined with pemetrexed–platinum	0.983 (0.639-1.512)			
combined with taxane–platinum	1.200 (0.749-1.920)			
Gene mutation type				
no genetic mutation	1.000	0.627		
EGFR exon 18 point mutation	0.607 (0.195-1.892)			
EGFR exon 19 deletion	1.089 (0.892-1.330)			
EGFR exon 20 insertion	0.985 (0.628-1.545)			
EGFR the L858R point mutation	0.889 (0.735-1.074)			
EGFR double mutation	1.093 (0.517-2.309)			
ALK rearrangement	1.025 (0.660-1.592)			
ROS1 rearrangement	0.308 (0.099-0.961)			
RET rearrangement	0.723 (0.299-1.749)			
BRAF mutation	1.226 (0.457-3.287)			
HER2 mutation	0.662 (0.295-1.483)			
KRAS mutation	0.856 (0.551-1.329)			
MET aberration	0.855 (0.354-2.068)			
Brain metastases				
with vs without	1.137 (0.958-1.349)	0.142		
Liver metastases				
with vs without	1.309 (1.077-1.592)	0.007	1.207 (0.990-1.470)	0.062
History of hypertension				
with vs without	1.205 (0.927-1.751)	0.030		

*p < 0.05.

### Safety

As shown in **Table 5**, the incidence of treatment-related grade AEs in all patients was 74.42% (704/946). Among which, 199 cases (21.04%) were more than grade 3 AEs. No fatal effects happened. Similar incidences of TEAEs at any grade were observed in the biosimilar and reference groups (421 subjects [76.41%] vs. 283 subjects [71.65%], *P*=0.098), and most of them were classified as grade 1 or 2 AEs. No statistically significant differences in the incidence of grade 3 and 4 TEAEs were observed between the two groups (22.14% vs. 19.49%, *P*=0.324). Among the treatment-related AEs in the biosimilar group, neutropenia had the highest incidence (22.87%), followed by anemia (16.15%), alopecia (14.70%), nausea (12.89%), fatigue (10.16%), thrombocytopenia (10.16%), hypertension (9.62%), and fever (8.71%). In the reference group, neutropenia had the highest incidence (24.05%), followed by anemia (16.46%), alopecia (13.67%), fatigue (11.90%), thrombocytopenia (10.89%), nausea (10.63%), loss of appetite (9.37%), and bleeding (7.34%). The incidence of hypertension at any grade in the biosimilar group was higher than that in the reference group (9.62% vs. 5.82%, *P*=0.033), but no difference in the incidence of hypertension was found at grade 3 or 4 (1.63% vs. 0.51%, *P*=0.093). We further analyzed the clinical data to understand the origin of the differences. Among patients over 70 years of age who received high-dose treatment, the incidence of hypertension caused by the biosimilar (n=48) was significantly higher than that of reference bevacizumab (n=32; 37.5% vs. 15.6%, *P*=0.034). Among patients over 70 years of age with a history of hypertension, the incidence of hypertension caused by the biosimilar (n=25) was higher than that of reference bevacizumab (n=17; 40% vs. 23.5%, *P*=0.266). Among these patients, we first hypothesized that differences in the incidence of hypertension might be attributed to some patients in the subgroup over 70 years of age. We then compared the incidence of hypertension in patients over 70 years of age after excluding patients with high-dose therapy and a history of hypertension. The results showed that the incidence of hypertension was similar between the two products (17.3% vs. 10.0%, *P*=0.511). After imbalanced factors were eliminated, no statistically significant difference in the incidence of hypertension of any grade was found between the biosimilar group (n=541) and reference group (n=392; 7.9% vs. 5.1%, *P*=0.087).

**Table 5:** Treatment-related adverse events.

	biosimilar group (n=551)		reference group (n=395)	
Adverse events	any grade	Grade 3-4	any grade	Grade 3-4
General disorders				
fatigue	56 (10.16%)	7 (1.27%)	47 (11.90%)	4 (1.01%)
fever	48 (8.71%)	18 (3.27%)	22 (5.57%)	9 (2.28%)
Infectious diseases				
pneumonia	7 (1.27%)	2 (0.36%)	3 (0.76%)	0 (0.00%)
Blood and lymphatic system disorders				
anemia	89 (16.15%)	2 (0.36%)	65 (16.46%)	2 (0.51%)
leucopenia	36 (6.53%)	3 (0.54%)	21 (5.32%)	4 (1.01%)
neutropenia	126 (22.87%)	67 (12.16%)	95 (24.05%)	42 (10.63%)
thrombocytopenia	56 (10.16%)	11 (2.00%)	43 (10.89%)	5 (1.27%)
Vascular disorders				
hypertension	53 (9.62%)*	9 (1.63%)	23 (5.82%)*	2 (0.51%)
bleeding	39 (7.08%)	5 (0.91%)	29 (7.34%)	4 (1.01%)
thromboembolism	3 (0.54%)	0 (0.00%)	2 (0.51 %)	0 (0.00%)
Urinary disorders				
creatinine increased	10 (1.81%)	2 (0.36%)	9 (2.28%)	6 (1.52%)
proteinuria	5 (0.91%)	3 (0.54%)	6 (1.52%)	2 (0.51%)
Respiratory disorders				
cough	2 (0.36%)	1 (0.18%)	0 (0.00%)	0 (0.00%)
Gastrointestinal disorders				
diarrhea	3 (0.54%)	0 (0.00%)	1 (0.25%)	1 (0.25%)
nausea	71 (12.89%)	6 (1.09%)	42 (10.63%)	3 (0.76%)
vomiting	34 (6.17%)	3 (0.54%)	17 (4.30%)	3 (0.76%)
intestinal obstruction	2 (0.36%)	0 (0.00%)	0 (0.00%)	0 (0.00%)
elevated ALT or AST	14 (2.54%)	6 (1.09%)	7 (1.77%)	3 (0.76%)
Nervous system disorders				
headache	13 (2.36%)	1 (0.18%)	11 (2.78%)	1 (0.25%)
paresthesia	3 (0.54%)	0 (0.00%)	1 (0.25%)	0 (0.00%)
Skin and subcutaneous tissue disorders				
hair loss	81 (14.70%)	2 (0.36%)	54 (13.67%)	1 (0.25%)
rash	7 (1.27%)	0 (0.00%)	3 (0.76%)	2 (0.51%)
Musculoskeletal connective tissue disorders				
joint pain	2 (0.36%)	1 (0.18%)	1 (0.25%)	0 (0.00%)
Metabolism and nutrition disorders				
poor appetite	42 (7.62%)	1 (0.18%)	37 (9.37%)	2 (0.51%)
**Total**	421 (76.41%)	122 (22.14%)	283 (71.65%)	77 (19.49%)

Number of patients with an event (percent). *p < 0.05; ALT, alanine aminotransferase; AST, aspartate transaminase.

## Discussion

The results of this study demonstrated that bevacizumab biosimilar is equivalent in efficacy to bevacizumab in locally advanced and advanced NSCLC. Patients receiving previous treatment, patients receiving regimens other than in combination with chemotherapy, patients with rare genetic mutations, and patients with brain metastases also benefit clinically from both products. The AE spectra and incidence rates of the two products were similar.

Bevacizumab is a humanized monoclonal antibody targeting VEGF and is prepared by recombinant DNA technology. By combining with VEGF, it can inhibit the binding of VEGF to its receptor and block the signaling pathway of angiogenesis in tumor tissues. Bevacizumab has become an important component of systemic therapy for advanced NSCLC without genetic mutations. A biosimilar is an approved biological product that is highly similar to the original drug with no clinically remarkable difference in safety, purity, or potency. Biosimilar therapy is an important avenue to reduce patient expenditure and the financial burden of national healthcare systems while maintaining therapeutic effect (18). However, a biosimilar is not an exact copy of its reference product and requires vigilance and concern in clinical application.

Current prospective clinical trials have confirmed that bevacizumab biosimilar in combination with platinum-containing two-drug chemotherapy has similar efficacy and safety compared with bevacizumab in patients with untreated advanced non-squamous NSCLC. In 2019, China National Medical Products Administration approved the marketing of bevacizumab biosimilar developed by Qilu Pharmaceutical Co., Ltd. (trade name: Encoda) with all indications approved for bevacizumab in China. However, no clinical data has verified the efficacy and safety of the biosimilar in clinical application. Existing trials have excluded patients receiving previous treatment, patients receiving combination with targeted therapy or immunotherapy, patients with brain metastases, patients with rare genetic mutations (such as EML4–ALK rearrangement), and patients with ECOG scores greater than 2. No consensus has been reached on the efficacy and safety of bevacizumab biosimilar in these populations. Among the published retrospective studies comparing bevacizumab biosimilar with bevacizumab, most have focused on patient clinical characteristics, cost-effectiveness, and economic impact rather than efficacy and safety. Only one study mentioned the statistical analysis of treatment modalities for 18 patients with NSCLC who used bevacizumab (19). Our study is not comparable to this one because of the lack of other information and its limited patient population. Our study greatly expanded the sample size and provided detailed information. It is the first retrospective study to evaluate the efficacy and safety of bevacizumab biosimilar.

Subject demographics, baseline disease characteristics. And exposure to treatment were well balanced and comparable between the two treatment groups. In the biosimilar group, the ORR was 28.9% (95% CI: 25.1%–32.7%), and the DCR was 89.5%. The ORR and DCR of the control group were 30.9% (95% CI: 26.3%–35.5%) and 87.3%, respectively. The unstratified ORR risk ratio was 0.934 with a 95% CI of 0.767–1.138 and a 90% CI of 0.792–1.103. The unstratified ORR risk difference was −0.020 with a 95% CI of −0.118–0.035 and a 90% CI of −0.105–0.023. The definition of equivalence by FDA, PMDA, and EMA corresponds to the 90% CI of ORR hazard ratio in the range of 0.73–1.37; the 95% CI of the ORR HR is 0.729–1.371, and the 95% CI of the ORR risk difference is −13%–13% (20, 21). In the present study, the CIs of ORR risk ratio and ORR risk difference were within these predefined equivalence margins. The estimated mPFS values were 6.27 (95% CI: 5.53–7.01) months in the biosimilar group and 4.93 (95% CI: 4.24–5.62) months in the reference group. The estimated HR for bevacizumab biosimilar and bevacizumab comparison was 1.084 (95% CI: 0.932–1.260, *P*=0.296). Therefore, bevacizumab biosimilar and reference bevacizumab are equivalent in efficacy.

In different historical clinical trial results for bevacizumab biosimilar, the ORR ranges from 41.5% to 53.1%, and the mPFS is about 7.5 months (22–24). Numerically, our study showed a lower ORR and a shorter PFS than other studies. In view of the differences between clinical application and clinical trials, such as the number of treatment lines, drug combinations, and dose intensity, the above results are not highly comparable. The subgroup analyses were performed based on the following subgroups: sex, age, pathology, stage, number of treatment lines, radiotherapy, combined treatment regimens, combined chemotherapy regimens (if combined with chemotherapy), dose intensity, genetic mutations, brain metastasis, liver metastasis, and history of hypertension. To avoid the increased risk of potential bias associated with a small sample size, subgroups with less than 3 cases were not included in the subgroup analysis.

In the subgroup analysis of combined treatment regimens, the ORR of the biosimilar group was lower than that of the reference group when combined with chemotherapy and immunotherapy (22.58% vs. 33.64%, *P*=0.048). Further logistic regression analysis showed that the influencing factors of ORR in patients combined with chemotherapy and immunotherapy were the number of treatment lines and combination with radiotherapy (*P*=0.021, 0.008). Product type (i.e., bevacizumab or bevacizumab biosimilar) was not a main factor (*P*=0.604). In a subgroup of patients with a history of hypertension, bevacizumab biosimilar obtained a longer PFS than reference bevacizumab (8.85 vs. 6.63 months, *P*=0.047). The mean dose intensity of the biosimilar group was significantly higher than that of the reference group (8.28 vs. 7.37 mg/kg, *P*=0.007) in this subgroup. Based on multivariate analysis, the product type was not the main factor affecting PFS (*P*=0.595). Among the remaining subgroups, the ORR and PFS analyses support the equivalence between the two groups.

The number of treatment lines, combined treatment regimens, and radiotherapy were the significant factors affecting the PFS of both groups (*P*<0.001, *P*=0.001, *P*=0.039). Combined chemotherapy regimens (if combined with chemotherapy), different genetic mutations, dose intensity, brain metastases, liver metastases, and a history of hypertension were not the main factors affecting PFS (*P*=0.104, 0.627, 0.946, 0.142, 0.062, 0.030).

The treatments combined with chemotherapy and another targeted therapy (biosimilar group: ORR=52.94%, mPFS=15.25 months; reference group: ORR=65.00%, mPFS=8.17 months) and combined with another targeted therapy group (biosimilar group: ORR=33.33%, mPFS=11.74 months; reference group: ORR=50.00%, mPFS=18.20 months) showed better efficacy than other treatment regimens. Among these treatments, approximately 80% of other targeted therapies are EGFR–TKI. Preclinical studies have shown that VEGF and EGFR share a common downstream signaling pathway (25–28), but the clinical trial data of EGFR–TKI combined with bevacizumab are still immature and have many uncertainties (29). Our study confirmed the benefits of bevacizumab (or bevacizumab biosimilar) combined with EGFR–TKI in ORR and PFS but failed to obtain OS results for all patients because of the short follow-up period. Previous studies have shown that patients with exon 19 deletions have a better prognosis after EGFR–TKI treatment than those with 21 p.L858 mutations (30). However, our study revealed similar ORR and PFS benefits between the two mutation types in both groups. The synergistic antiproliferative effects of EGFR–TKI and antiangiogenic treatment might eliminate the prognostic differences caused by genetic mutations. More prospective studies or in-depth retrospective clinical data are expected to better explore the above conclusions.

In addition to EGFR mutations, studies on patients with other rare mutations receiving targeted therapy combined with bevacizumab are few, and all of which are exploratory studies with small samples. The American Society of Clinical Oncology reported a study from China in which 16 patients with EML4–ALK rearrangement receiving crizotinib and bevacizumab have a mPFS of 13.0 months. Our study included 89 patients that harbor other rare driving gene mutations, including ALK rearrangement, ROS1 rearrangement, RET rearrangement, BRAF mutation, HER2 mutation, KRAS mutation, and MET aberration. These populations have also been proven to benefit from bevacizumab or bevacizumab biosimilar.

In the subgroup analysis of combined with chemotherapy, multivariate analysis showed the combined chemotherapy regimen was not the main factor affecting PFS (*P*=0.068). But it is worth noting that, numerically, the treatment combined with gemcitabine showed the shortest PFS and the treatment combined with pemetrexed–platinum showed the longest PFS in both the biosimilar and reference groups (biosimilar group: pemetrexed vs taxane vs gemcitabine vs pemetrexed–platinum vs taxane–platinum: 3.17m vs 3.20m vs 1.15m vs 4.43m vs 3.41m, *P*=0.119; reference group:1.97m vs 2.27m vs 1.59m vs 4.23m vs 3.57m, *P*=0.055). Previous studies (14) have confirmed that gemcitabine is inferior to pemetrexed or paclitaxel in advanced NS-NSCLC patients. Our results suggest that this trend may remain in the context of bevacizumab in combination. Whether the efficacy of bevacizumab in combination with different platinum-based doublets is different is still controversial. PointBreak trial (31) confirmed that pemetrexed–platinum combined with bevacizumab regimen obtained significantly longer PFS than taxane–platinum combined with bevacizumab regimen, but no statistical difference was observed in PRONOUNCE trial (32)and ERACLE trial (33). In our study, no statistical difference was obtained on this question, probably due to the inherent influence of selection bias and missing data. Larger prospective studies are expected to investigate this issue.

In the previous phase III clinical trial studies, only the AVAiL and AVAPERL studies used bevacizumab at 7.5 mg/kg, and the other studies used 15 mg/kg. These studies lacked the ability to directly compare the two doses of bevacizumab. With 7.5 mg/kg as the boundary line, our study showed that no statistical differences in the ORR and PFS between the low- and high-dose groups (biosimilar group: 33.19% vs. 25.85%, 6.70 vs. 5.90 months; reference group: 29.79% vs. 32.50%, 4.80 vs. 5.00 months). In addition, the patients with the brain metastases (BMS) also benefit from bevacizumab or biosimilar (biosimilar group: ORR=26.67%, mPFS=6.07 months; reference group: ORR=23.60%, mPFS=4.43 months).

Similar incidences of TEAEs at any grade were observed in the biosimilar group and the reference group (421 [76.41%] vs. 283 subjects [71.65%], *P*=0.098), and most of them were classified as grade 1 or 2 events. No statistically significant difference in the incidence of grade 3 or 4 TEAEs was observed between the two groups (22.14% vs. 19.49%, *P*=0.324). No fatal effects happened. Among the treatment-related AEs in the biosimilar group, neutropenia had the highest incidence (22.87%), followed by anemia (16.15%), alopecia (14.70%), nausea (12.89%), fatigue (10.16%), thrombocytopenia (10.16%), hypertension (9.62%), and fever (8.71%). In the reference group, neutropenia had the highest incidence (24.05%), followed by anemia (16.46%), alopecia (13.67%), fatigue (11.90%), thrombocytopenia (10.89%), nausea (10.63%), loss of appetite (9.37%), and bleeding (7.34%). In general, the AE spectra and AE rates of bevacizumab biosimilar and reference bevacizumab were similar. The overall incidences of grade 3 and 4 TEAEs were low and similar, indicating that bevacizumab biosimilar and bevacizumab have favorable safety profiles.

The incidence of hypertension at any grade in the biosimilar group was higher than that in the reference group (9.62% vs. 5.82%, *P*=0.033), but no differences in the incidence of hypertension were observed at grades 3 and 4 (1.63% vs. 0.51%, *P*=0.093). We further analyzed the clinical data to understand the origin of the differences. Among patients over 70 years of age who received high-dose treatment, the incidence of hypertension caused by the biosimilar (n=48) was significantly higher than that of reference bevacizumab (n=32; (37.5% vs. 15.6%, *P*=0.034). Among patients over 70 years of age with a history of hypertension, the incidence of hypertension caused by biosimilar (n=25) was higher than that of reference bevacizumab (n=17; 40% vs. 23.5%, *P*=0.266). Among these patients, we first hypothesized that differences in the incidence of hypertension might be attributed to some patients in the subgroup over 70 years of age. We then compared the incidence of hypertension in patients over 70 years of age after excluding patients with high-dose therapy and a history of hypertension. The results showed that the incidence of hypertension was similar between the two products (17.3% vs. 10.0%, *P*=0.511). After imbalanced factors were eliminated, no statistically significant difference in the incidence of hypertension of any grade was found between the biosimilar group (n=541) and reference group (n=392; 7.9% vs. 5.1%, *P*=0.087).

In the subgroup analysis of combined with chemotherapy, similar incidences of TEAEs at any grade were observed (pemetrexed vs taxane vs gemcitabine vs pemetrexed–platinum vs taxane–platinum: 69.2% vs 78.3% vs 55.6% vs 71.9% vs 72.6%, *P*=0.695). Among these treatment-related AEs, neutropenia had the highest incidence (28.92%), followed by anemia (18.92%), thrombocytopenia (11.54%) and fatigue (10.77%). The incidence of a few adverse reactions varied with chemotherapy regiments, and these differences existed in both the biosimilar and reference groups. The incidence of anemia at any grade was significantly higher in the treatment combined with pemetrexed–platinum than in the other chemotherapy regimens (biosimilar group:10.0% vs 13.3% 0.00% vs 26.8% vs 12.1%, *P*=0.042; reference group:12.5% vs 13.3% vs 0.00% vs 23.1% vs.10.8%, *P*=0.039). All 4 cases of sensory neuropathy were in the taxane–platinum combined with bevacizumab regimen (biosimilar group vs reference group: 5.2% vs 2.7%, *P*=0.654), but all were classified as grade 1 or grade 2 events. The above adverse reactions were consistent with previous studies on corresponding chemotherapy regimens. These were not attributed to the type of bevacizumab product. The toxicity of these adverse reactions is considered acceptable, but close monitoring of these patients is still required in clinical practice.

We acknowledge the limitations of the retrospective study design, which is inherently affected by selection bias and missing data. Moreover, reliance on electronic health records may mean that some events may be underestimated. Therefore, larger prospective studies are needed to confirm our findings.

## Conclusions

The results of this study demonstrated that bevacizumab biosimilar is equivalent in efficacy to bevacizumab in patients with locally advanced and advanced NSCLC. Bevacizumab biosimilar showed acceptable toxicity profile and no new AEs. Patients receiving previous treatment, patients receiving regimens other than in combination with chemotherapy, patients with rare genetic mutations, and patients with brain metastases can also benefit clinically from both products. The number of treatment lines, radiotherapy, and combined treatment regimens were the substantial factors affecting the ORR and PFS of bevacizumab or biosimilar. Different genetic mutations and dose intensity were not the main factors affecting PFS.

## Data availability statement

The raw data supporting the conclusions of this article will be made available by the authors, without undue reservation.

## Ethics statement

The studies involving human participants were reviewed and approved by Academic Ethics Committee of Jiangsu Cancer Hospital. Written informed consent for participation was not required for this study in accordance with the national legislation and the institutional requirements.

## Author contributions

All authors listed have made a substantial, direct, and intellectual contribution to the work, and approved it for publication.
